# Air pollution and male infertility: A meta-umbrella study of evidence on semen quality

**DOI:** 10.1097/MD.0000000000047806

**Published:** 2026-03-06

**Authors:** Ria Margiana, Maryam Abdulrahman Najim, Aumaima Tariq Abed, Samer Saleem Alshkarchy, Patricio Yáñez-Moretta, K.D.V. Prasad, Juan Jara-Santamaría, Zahraa Abbas Khafaji, Amirali Ebrahimi

**Affiliations:** a Department of Anatomy, Faculty of Medicine, Universitas Indonesia, Jakarta, Indonesia; b Master’s Programme Biomedical Sciences, Faculty of Medicine, Universitas Indonesia, Jakarta, Indonesia; c Universitas Indonesia Academic Hospital, Depok, Indonesia; d Permata Depok Hospital, Depok, Indonesia; e Twins Fertility Clinic, Jakarta, Indonesia; f Bocah Indonesia Fertility Center, Jakarta, Indonesia; g Harapan Kita Hospital, Jakarta, Indonesia; h Department of Biology, College Education for Pure Science, University of Anbar, Al-Anbar, Iraq; i Department of Pharmacy, Al-Zahrawi University College, Karbala, Iraq; j Medical Laboratory Techniques Department, Health And Medical Technique College, University of Hilla, Babylon, Iraq; k Department of Pathology, Al-Qasim Green University, Hilla, Iraq; l Escuela de Ciencias Biológicas, Universidad de Investigación de Tecnología Experimental Yachay, Urcuquí, Ecuador; m Symbiosis Institute of Business Management, Hyderabad, Symbiosis International (Deemed University), Pune, India; n Metroimagen SAS, Latacunga, Ecuador; o College of Pharmacy, The Islamic University, Najaf, Iraq; p Guilan University of Medical Sciences, Rasht, Iran.

**Keywords:** air pollution, male infertility, meta-analysis, meta-umbrella, semen quality

## Abstract

**Background:**

Growing epidemiological evidence links air pollution to male reproductive health through oxidative stress, endocrine disruption, and direct cellular damage. However, the magnitude and consistency of effects on semen quality parameters across different pollutant types and exposure durations remain uncertain.

**Methods:**

We systematically searched PubMed, Web of Science, and Scopus through January 2025 for meta-analytic studies evaluating the impact of air pollution on semen parameters. Four eligible studies were assessed for methodological quality using AMSTAR 2. Random-effects meta-analyses yielded standardized mean differences (SMDs) for sperm concentration, count, total motility, progressive motility, and morphology.

**Results:**

We observed statistically significant negative associations between higher air pollution exposure and semen quality outcomes. Specifically, greater pollutant exposure was associated with lower total motility (SMD ‐0.24; 95% CI ‐0.48 to ‐0.01; *P* = .04), progressive motility (SMD ‐0.06; 95% CI ‐0.10 to ‐0.01; *P* < .001), and morphology (SMD ‐0.09; 95% CI ‐0.13 to ‐0.04; *P* < .001). Concentration was inversely related to pollution levels (SMD ‐0.14; 95% CI ‐0.21 to ‐0.06; *P* < .001), while sperm count showed a trend toward reduction but did not reach statistical significance (SMD ‐0.12; 95% CI ‐0.26 to 0.01; *P* = .06).

**Conclusion:**

This meta-umbrella analysis confirms consistent, modest negative associations between ambient air pollution and key semen quality parameters, underscoring air pollution as a modifiable risk factor for male infertility. Future studies should define threshold exposures and evaluate protective interventions.

## 1. Introduction

Air pollution has become one of the hottest topics in recent years due to its adverse effects on human health.^[1,2]^ Previous studies showed the impact of air pollution on the cardiovascular system, respiratory system, and cancers.^[3,4]^ Previous studies have demonstrated that exposure to air pollution is associated with decreased semen quality, including reductions in sperm concentration, motility, and normal morphology.^[5]^

Air pollution complications can be more pronounced in certain areas due to the development and industrialization of several cities, traffic congestion, automobile exhaust, and the burning of industrial waste.^[5]^ A recent study showed that male fertility is decreasing in industrial countries, and increasing air pollution is contributing to this.^[6]^

Air pollution encompasses a complex mixture of harmful substances, including particulate matter (PM) (PM2.5 and PM10), nitrogen dioxide (NO_2_), sulfur dioxide, carbon monoxide, ozone, and polycyclic aromatic hydrocarbons (PAHs).^[7]^ These pollutants can become concentrated in certain geographical areas due to rapid industrialization, urban development, traffic congestion, automobile exhaust emissions, and the burning of industrial waste. Recent epidemiological evidence demonstrates that male fertility rates are declining in industrialized countries, with increasing air pollution contributing significantly to this concerning trend.^[8–11]^

One of the critical components of the male reproductive system is semen quality, which depends on sperm concentration, semen volume, sperm count, total motility, progressive motility, and normal morphology.^[12]^

Accumulating research evidence reveals that air pollution exposure adversely affects semen quality through several pathways.

Exposure to PM (PM2.5 and PM10) has been consistently associated with decreased sperm concentration, reduced total and progressive motility, and increased risk of asthenozoospermia. NO_2_ exposure specifically correlates with reduced total sperm number and increased risk of semen volume abnormalities. Studies have demonstrated that sulfur dioxide exposure leads to decreased sperm motility and reduced total sperm count across multiple exposure windows during spermatogenesis.^[7–9,13]^ Watanabe et al demonstrated a reduction in sperm volume following exposure to diesel exhaust during fetal development. It also demonstrated that exposure to certain air pollutants, such as PAHs, can impair sperm function by inducing deoxyribonucleic acid (DNA) damage and oxidative stress, thereby compromising male fertility potential.^[14]^

PAHs, prevalent components of air pollution from combustion processes, induce oxidative stress and cause sperm DNA fragmentation, compromising genetic integrity and potentially affecting offspring health.

The mechanisms underlying these effects involve increased reactive oxygen species (ROS) production, lipid peroxidation of sperm membranes, DNA damage through oxidative stress, and disruption of mitochondrial function in spermatozoa.

Additionally, air pollutants can alter sperm DNA methylation patterns and affect reproductive hormone levels, further impacting male fertility potential.

The reproductive consequences of air pollution exposure can be particularly severe due to the extended 90-day spermatogenesis cycle, during which developing sperm remain vulnerable to environmental toxicants. Research indicates that different phases of sperm development exhibit varying susceptibility to specific pollutants, with early spermatogenesis being more vulnerable to particulate matter exposure than later developmental stages.^[13,15,16]^

Male infertility imposes substantial socioeconomic burdens alongside personal health consequences, including reduced labor force participation and increased healthcare costs.^[12]^ The long-term societal implications of declining male fertility underscore the critical importance of identifying and controlling environmental risk factors. Although previous studies have established associations between air pollution and male infertility, the precise quantitative effects on specific semen parameters remain inconsistent across different populations and geographical regions. Previous meta-analyses have reported conflicting results regarding the magnitude of these effects.^[17,18]^ Therefore, we aimed to conduct a comprehensive meta-umbrella study to systematically evaluate the impact of air pollution on semen quality parameters based on current evidence-based knowledge and provide definitive conclusions regarding these associations.

## 2. Methods

The present study is a meta-umbrella study of the effect of air pollution on male reproductive health. The study was conducted in 2025. The Preferred Reporting Items for Systematic Review and Meta-analysis guideline was used for the study protocol.^[19]^

### 2.1. Literature search

A comprehensive systematic search was performed on PubMed, Web of Science, and Scopus databases from inception until January 2025. The search strategy was developed using a combination of free-text keywords and controlled vocabulary terms (MeSH for PubMed, Emtree for Embase, etc, adapted for each database). The core concepts included “air pollution,” “male infertility,” and “semen quality.” The following is an example of the search string used for PubMed:

(“Air Pollution”[Mesh] OR “Air Pollutants”[Mesh] OR “Particulate Matter”[Mesh] OR “Environmental Exposure”[Mesh] OR “air pollut*”[tiab] OR “PM2.5”[tiab] OR “nitrogen dioxide”[tiab] OR “NO_2_”[tiab])

AND

(“Infertility, Male”[Mesh] OR “male infertil*”[tiab] OR “semen quality”[tiab] OR “sperm motility”[tiab] OR “sperm count”[tiab] OR “sperm concentration”[tiab] OR “sperm morphology”[tiab] OR “azoospermia”[tiab] OR “oligospermia”[tiab])

This strategy was adapted for syntax and available controlled vocabularies in Web of Science and Scopus. The reference lists of all included studies and relevant reviews were also manually screened to identify any additional eligible publications.

### 2.2. Study selection and eligibility criteria

We included meta-analysis studies with the following criteria:

1. The population of the study was male patients.
2. Studies that assessed air pollution effects on the male reproductive system.
3. At least one outcome related to semen quality was considered.
4. Studies in English are available in full text. Narrative reviews, editorials, commentaries, and randomized clinical trials were excluded.

### 2.3. Data extraction

We extracted the following data from included studies: first author name, year of publication, searching databases, date of search, the final number of included studies and several patients, and any outcome on semen quality.

### 2.4. Quality evaluation

The AMSTAR2 checklist was used for evaluating the included studies.^[20]^ This checklist consists of 16 questions assessing the methodological quality of systematic review studies.^[20]^

### 2.5. Statistical analyses

We analyze the results using Comprehensive Meta-Analysis software version 3 (Biostat, Inc., Englewood). The included studies’ effect size and 95% confidence interval were examined to determine the overall effect. We chose the between-study heterogeneity by *I*^2^ statistics and Cochrane *Q*-test. High heterogeneity was observed when *I*^2^ >50% and *P*-value <0.1. We used the random effect model when heterogeneity existed; otherwise, a fixed effect model was used. We also performed a sensitivity analysis to assess the effect of every single study on the overall impact. A power analysis was also conducted for each outcome. Sensitivity analyses were also conducted to evaluate the robustness of the results by systematically excluding individual studies.

## 3. Results

**Figure 1.**
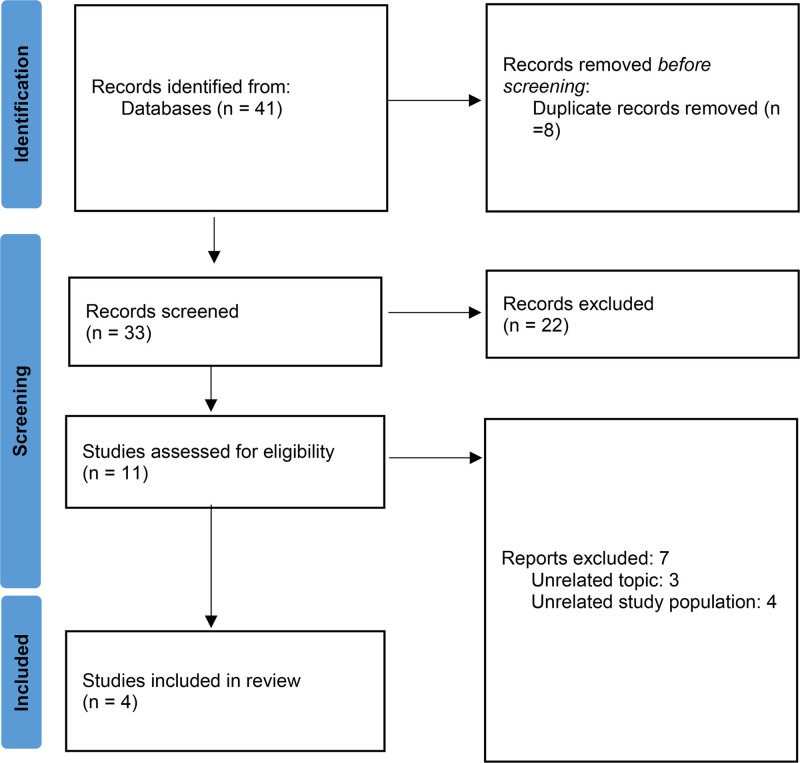
PRISMA flow chart of study selection. PRISMA = Preferred Reporting Items for Systematic Review and Meta-analysis.

The first literature search resulted in 41 studies. After removing duplication, 33 studies were left. We studied the remaining articles by title, abstract, and full text, and 22 studies were eliminated for various reasons (15 of them were excluded because they had unrelated study populations, and from the remaining 7 studies, 5 of them had unrelated topics, and the other 2 were not meta-analyses). Finally, 4 meta-analyses were left assessing the effect of air pollution on semen quality. Flowchart of the included eligible studies presented in Figure 1. The characteristics of the included studies are shown in Table 1.

**Table 1:** Characteristics of included studies.

References	Journal	Databases	Date of search	The final number of included studies	Number of total patients	The mean age of the participants	Statistical model	Statistical software	Variables
Pizzol et al^[21]^	Environmental Science and Pollution Research	MEDLINE/PubMed, Scopus, CINAHL, Embase, PsycINFO, and Cochrane Library	From inception up to March 31, 2020	22	4460	30.8 ± 6.8	Random effect	Stata	Sperm volume, sperm count, sperm concentration, sperm vitality, sperm motility, sperm morphology, sperm DNA fragmentation, chromatin damage
Deng et al^[12]^	Environmental Pollution	PubMed and Web of Science	From inception up to May 30 2015	6	1979	Not reported	Random effect	R software	Sperm volume, sperm concentration, sperm count, sperm progressive motility, sperm total motility, sperm morphology
Qian et al^[22]^	Environmental Science and Pollution Research International	PubMed, Web of Science Core Collection, and Cochrane Library	From inception up December 2019	6	4465	Not reported	Random effect model for heterogenic and fixed effect model for non-heterogenic studies	Stata	Sperm concentration, sperm count, sperm total motility, sperm progressive motility
Zhang et al^[23]^	BioMed Research International	PubMed, Web of Science, and Embase		11	4562	Not reported	Random effect	Not reported	Semen volume, sperm concentration, sperm count, sperm progressive motility, sperm total motility, sperm morphology, sperm DNA fragmentation

DNA = deoxyribonucleic acid.

### 3.1. Subgroup analysis

#### 3.1.1. Semen volume

**Figure 2.**
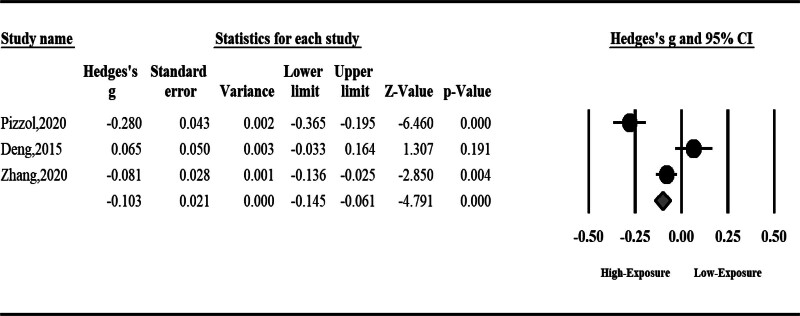
The effect of air pollution on semen volume.

Air pollution can reduce semen volume; however, the total effect was nonsignificant (standardized mean difference [SMD]: ‐0.10, 95% CI: ‐0.27 to 0.07, *P* = .25). The result was accompanied by high heterogeneity; hence, we used a random effect model (*I*^2^ = 93%, *P* < .001) (Fig. 2).

#### 3.1.2. Sperm total motility

**Figure 3.**
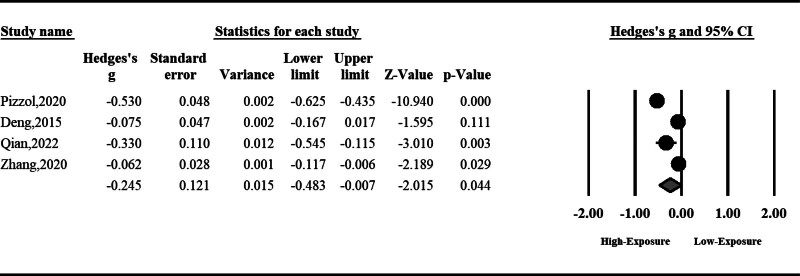
The effect of air pollution on sperm total motility.

Air pollution can significantly reduce sperm total motility (SMD: ‐0.24, 95% CI: ‐0.48 to ‐0.01, *P* = .04). The result was accompanied by high heterogeneity; hence, we used a random effect model (*I*^2^ = 96%, *P* < .001) (Fig. 3).

#### 3.1.3. Sperm progressive motility

**Figure 4.**
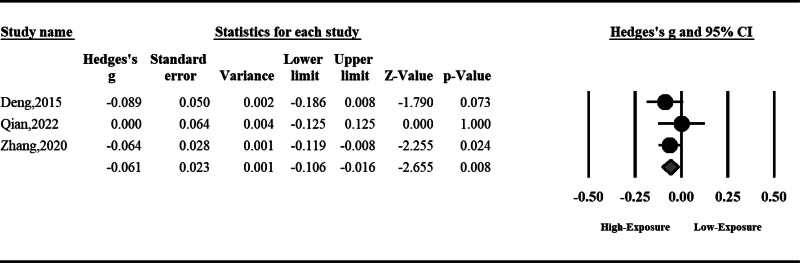
The effect of air pollution on sperm progressive motility.

Sperm progressive motility can be reduced significantly by air pollution (SMD: ‐0.06, 95% CI: ‐0.10 to ‐0.01, *P* < .001). No significant heterogeneity was observed among included studies; hence, the fixed effect model was used (Fig. 4).

#### 3.1.4. Sperm morphology

**Figure 5.**
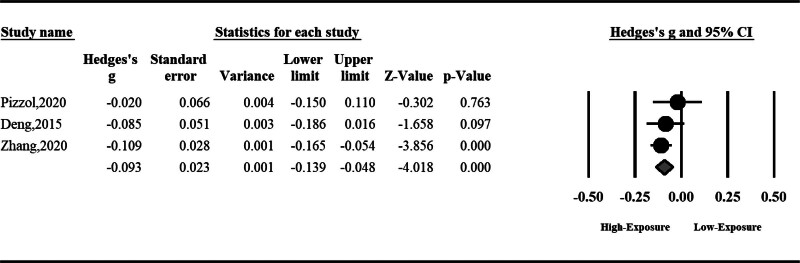
The effect of air pollution on sperm morphology.

Air pollution can significantly disturb sperm morphology (SMD: ‐0.09, 95% CI: ‐0.13 to ‐0.04, *P* < .001). No significant heterogeneity was observed among included studies; hence, the fixed effect model was used (Fig. 5).

#### 3.1.5. Sperm count

**Figure 6.**
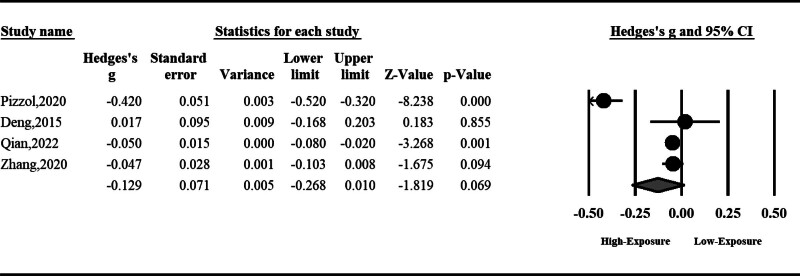
The effect of air pollution on sperm count.

Sperm count can be reduced by air pollution; however, the total effect was insignificant (SMD: ‐0.12, 95% CI: ‐0.26 to 0.01, *P* = .06). The result was accompanied by high heterogeneity; hence, we used a random effect model (*I*^2^ = 96%, *P* < .001) (Fig. 6).

#### 3.1.6. Sperm concentration

**Figure 7.**
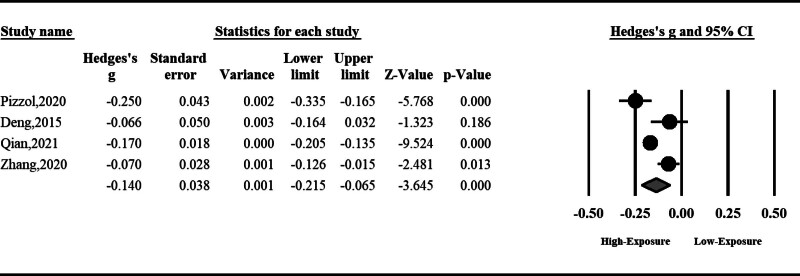
The effect of air pollution on sperm concentration.

Sperm concentration can be reduced significantly by air pollution (SMD: ‐0.14, 95% CI: ‐0.21 to ‐0.06, *P* < .001). The result was accompanied by high heterogeneity; hence, we used a random effect model (*I*^2^ = 82%, *P* < .001) (Fig. 7).

#### 3.1.7. Power analysis

**Figure 8.**
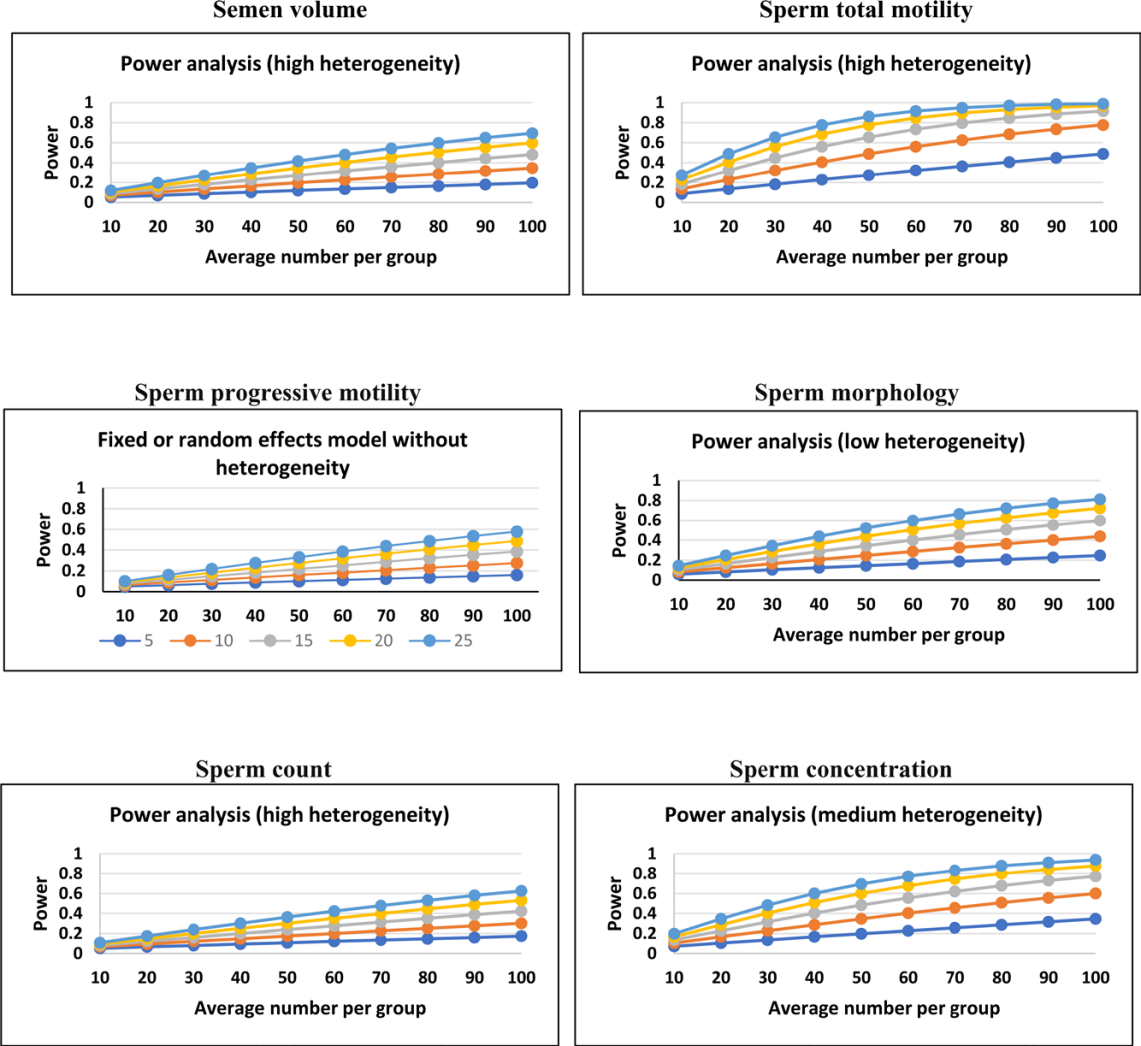
A power analysis to evaluate the different outcomes, including: semen volume, sperm total motility, sperm progressive motility, sperm morphology, sperm count, sperm concentration.

A power analysis was conducted utilizing Hedges’ g statistic to assess each of the outcomes examined. (semen volume = 0.8; sperm total motility = 1.0; sperm progressive motility = 0.91; sperm morphology = 0.99; sperm count = 0.98; sperm concentration = 0.99) (Fig. 8).

#### 3.1.8. Risk of bias assessment

We used the AMSTAR 2 appraisal checklist to evaluate the quality of studies. The result of the quality assessment is shown in File S1, Supplemental Digital Content, https://links.lww.com/MD/R435.

### 3.2. Sensitivity analysis

**Figure 9.**
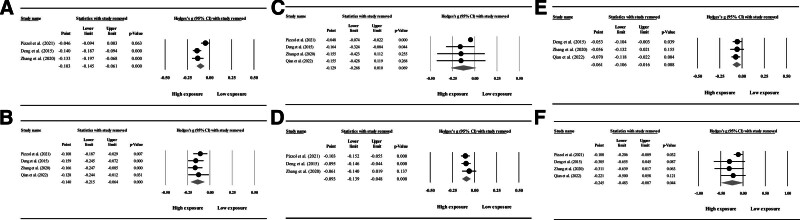
Sensitivity analysis with the “one study removal” method. (A) Semen volume, (B) sperm concentration, (C) sperm count, (D) sperm morphology, (E) sperm progressive motility, and (F) sperm total motility.

To account for the heterogeneity observed in our results, we performed a sensitivity analysis employing the “one study removal” approach to pinpoint potential sources of variability in assessing different parameters (semen volume, sperm total motility, sperm progressive motility, sperm morphology, sperm count, and Sperm concentration) (Fig. 9).

## 4. Discussion

The increasing trend of air pollution worldwide and its impact on various human health problems have resulted in a significant burden of cardiovascular,^[13]^ respiratory,^[14]^ and reproductive diseases,^[15]^ as well as cancers. The primary sources of air pollution are the exhaust of motor vehicles, waste treatment, oil refineries, fire, and factories.^[24]^ The main components of air pollutants that impact human health include particulate matter, nitrogen oxides, sulfur dioxide, ozone, volatile organic compounds, carbon monoxide, PAHs, and radiation. Serious health problems of air pollution can originate from the particles present in the air in the form of small liquid or solid droplets, which can enter the human body through inhalation.^[24]^ Moreover, particles <10 μm (PM_10_) are hazardous and can reach the bloodstream, causing various health conditions. Finer particles, like PM_2.5_, are even more harmful and cause more significant health risks.^[25]^

Male infertility has become a significant health problem in the last few years, along with the increasing worldwide infertility rate.^[24]^ One of the determinants of male fertility is semen quality, which can be affected by environmental pollution. The disqualification of semen by environmental pollution can impair sperm functions, spermatogenesis, and steroidogenesis.^[24]^

The present meta-umbrella study consolidates and extends the growing body of evidence demonstrating that air pollution exerts a detrimental impact on male reproductive health, particularly semen quality parameters. Our findings reveal significant reductions in sperm total motility, progressive motility, morphology, and concentration associated with exposure to ambient air pollutants. These results align with prior meta-analyses and epidemiological studies that have reported similar associations, underscoring the robustness and consistency of this relationship across diverse populations and geographic regions. The observed impairments in semen quality parameters are clinically relevant, given their established roles as predictors of male fertility potential and reproductive outcomes.

Different air pollutants can cause semen disqualification. Wang et al showed that sulfur dioxide and NO_2_ exposure can damage spermatogenesis, especially in the initial phase.^[26]^ Heavy metals such as lead, cadmium, and mercury, often from environmental exposures including air pollution, accumulate in male reproductive organs and impair semen quality by inducing oxidative stress, disrupting the hypothalamic–pituitary–gonadal axis, and causing sperm DNA damage.^[27]^ Ozone is another component that can cause a reduction in normal morphologic sperm.^[28]^ PM_2.5_ is dangerous to total sperm count,^[29]^ sperm head morphology,^[29,30]^ sperm concentration,^[31]^ sperm motility,^[32]^ and overall semen quality.^[33,34]^

The exact pathophysiology of how air pollutants can damage semen quality is unknown. However, some postulated mechanisms are as below: hormonal disruption, increased production of ROS, and sperm DNA alteration.^[12,21–23]^

Airborne pollutants interact with the male reproductive system through multiple synergistic pathways, with endocrine disruption emerging as a primary mechanism.^[7]^ Some components, lead, zinc, copper, and cadmium, present as heavy metals in the polluted air, have antiandrogenic, estrogenic, and antiestrogenic characteristics^[35,36]^ that cause disturbances in gametogenesis processes.^[33,37]^ Moreover, small particles like PM_2.5_ can be accumulated in reproductive organs, causing hormonal alterations resulting in Infertility (inhibiting testosterone binding to androgen receptors).^[7,38]^ This interference disrupts the hypothalamic–pituitary–gonadal axis, reducing luteinizing hormone (LH) pulsatility and impairing Leydig cell steroidogenesis. A dose–response relationship has been documented, with each 5 μg/m^3^ increase in PM2.5 correlating with a 2.3% decrease in serum testosterone levels.^[7]^

The prooxidant nature of air pollution initiates a cascade of ROS generation that overwhelms seminal antioxidant defenses. Animal studies have shown that pollutant particles can have different ROS activity based on their source of production and chemical structure.^[39]^ Particle matter in the air can carry ROS in addition to components with redox-active properties, which can produce ROS by infusing different organs.^[40]^ The oxidative stress leads to cellular damage,^[41]^ sperm DNA fragmentation,^[42]^ and lipid peroxidation.^[33,43]^

Furthermore, PAHs mimic endogenous estrogens through aryl hydrocarbon receptor activation, skewing the estrogen-to-androgen ratio and inducing apoptotic signaling in developing germ cells.^[7]^ Previous studies demonstrated that some particles, like PAHs, can alter DNA methylation and gene expression in spermatogenesis, resulting in male infertility.^[33,44]^ These hormonal perturbations create a hostile microenvironment for spermatogenesis, particularly affecting the delicate sertoli cell–germ cell interactions necessary for sperm maturation.

The subclinical nature of pollution-induced spermatotoxicity necessitates novel diagnostic approaches. Seminal oxidative stress biomarkers like 8-isoprostane and γ-glutamyltransferase show promise as exposure indicators, with recent trials demonstrating 89% sensitivity in distinguishing high-pollution cohorts. Antioxidant supplementation (N-acetylcysteine 600 mg/day, vitamin E 400 IU/day) partially mitigates motility impairments, though benefits plateau after 3 months, suggesting adjuvant therapies are needed. For severe cases, advanced sperm selection techniques using magnetic-activated cell sorting to eliminate DNA-fragmented sperm improve ICSI outcomes by 22%.^[7]^

### 4.1. Study limitations and methodological considerations

While this meta-umbrella study benefits from rigorous methodology, several limitations warrant consideration. Heterogeneity in exposure assessment methods across primary studies (questionnaires vs monitoring data) may introduce measurement bias. The observational nature of the included data precludes causal inference, though Mendelian randomization studies using pollution-associated genetic variants could strengthen etiological claims. Furthermore, the exclusion of non-English studies and potential publication bias toward significant results may inflate estimated effect sizes.

## 5. Conclusion

These findings demonstrate consistent, modest negative associations between ambient air pollution and key semen quality parameters but do not establish causality. While higher pollutant exposure correlates with lower sperm concentration, motility, and morphology, further longitudinal and mechanistic studies are needed to determine causal pathways and thresholds of harm.

## Author contributions

**Conceptualization:** Ria Margiana, Amirali Ebrahimi.

**Data curation:** K.D.V Prasad.

**Formal analysis:** K.D.V Prasad.

**Investigation:** Maryam Abdulrahman Najim, Samer Saleem Alshkarchy.

**Methodology:** Aumaima Tariq Abed, Samer Saleem Alshkarchy, Patricio Yáñez-Moretta.

**Project administration:** Juan Jara-Santamaría.

**Resources:** Zahraa Abbas Khafaji.

**Software:** Juan Jara-Santamaría.

**Supervision:** Patricio Yáñez-Moretta.

**Validation:** Zahraa Abbas Khafaji.

**Writing – original draft:** Ria Margiana, Maryam Abdulrahman Najim, Aumaima Tariq Abed, Zahraa Abbas Khafaji, Amirali Ebrahimi.

**Writing – review & editing:** Ria Margiana, K.D.V. Prasad, Juan Jara-Santamaría, Amirali Ebrahimi.

## Supplementary Material


