# Supplementary material for: Air pollution and male infertility: A meta-umbrella study of evidence on semen quality
Source: Medicine (Baltimore). 2026 Mar 6;105(10):e47806. doi: 10.1097/MD.0000000000047806 (PMC12975254; doi:10.1097/MD.0000000000047806)

Supplementary file 1. Quality assessment of included studies

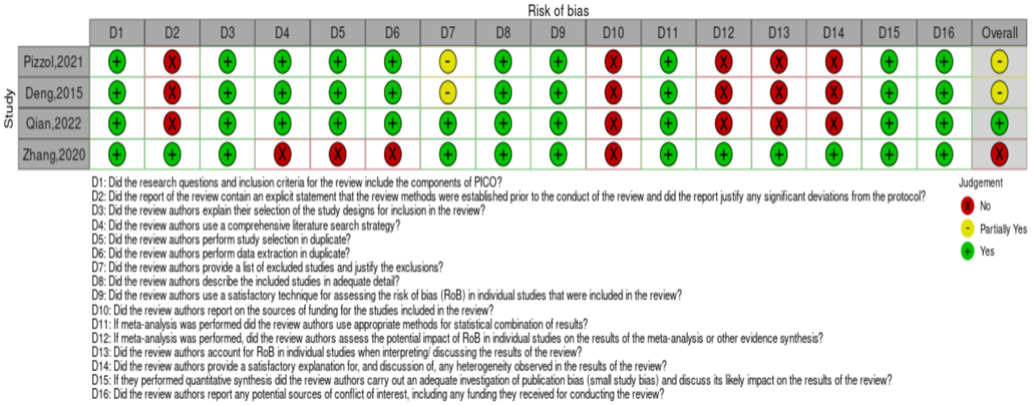

Supplement: Supplementary file 1 [file medi-105-e47806-s001.pdf]
